# Case Report: Redo carotid endarterectomy with patch angioplasty for treatment of restenosis caused by excessive intimal hyperplasia following endarterectomy: illustrative case

**DOI:** 10.3389/fsurg.2026.1754640

**Published:** 2026-02-26

**Authors:** Lu Zhao, Xinyu Wu, Wei Zhen, Fuyong Li

**Affiliations:** 1 First Department of Endocrinology, The People’s Hospital of Liaoning Province, Shenyang, Liaoning, China; 2 Third Department of Neurosurgery, The People’s Hospital of Liaoning Province, Shenyang, Liaoning, China

**Keywords:** carotid endarterectomy, case report, intimal hyperplasia, patch angioplasty, restenosis

## Abstract

**Introduction:**

Carotid endarterectomy (CEA) is considered the surgical intervention of choice for symptomatic and asymptomatic carotid artery stenosis. Restenosis following CEA is not a rare condition. However, cases of restenosis resulting from short-term massive intimal hyperplasia of the carotid artery are relatively rare.

**Case description:**

We present a case of a 69-year-old male patient who successively underwent carotid artery stenting (CAS), CEA and stent removal due to recurrent ischemic symptoms. Subsequently, the patient received redo carotid endarterectomy (reCEA) combined with patch angioplasty to address a third episode of carotid artery stenosis caused by extensive intimal hyperplasia. Based on a review of the relevant literature, the underlying pathological conditions and corresponding surgical strategies were analyzed and discussed.

**Conclusion:**

Symptomatic restenosis caused by simple intimal hyperplasia shortly following CEA is relatively uncommon. In contrast to atherosclerotic plaques, this dense and fibrous tissue is more resistant to dissection and may lead to a reduction in vessel diameter. Neither standard CEA nor CAS alone can adequately prevent long-term restenosis. However, CEA combined with patch angioplasty has been shown to be an effective therapeutic option for this specific type of stenosis.

## Introduction

For years, redo carotid endarterectomy (CEA) has been the standard treatment for post-CEA stenosis (1, 2), indications for carotid reintervention included patients with ≥50% symptomatic or ≥ 70% asymptomatic (including progressive carotid stenosis) post-CEA stenosis (1–5). With the advancement of interventional techniques, numerous studies have demonstrated favorable outcomes associated with CEA and CAS as therapeutic options for carotid restenosis (6–10). Carotid restenosis is the result of intimal hyperplasia in the early postoperative period (within 2 or 3 years) or recurrent atherosclerotic lesions at a later date (2, 4, 5, 11, 12). Currently, case reports regarding symptomatic restenosis caused solely by intimal hyperplasia remain relatively rare. While numerous studies have focused on the outcomes and risk factors associated with restenosis surgery, few provide detailed descriptions of the pathological changes in the intima following CEA. Many patients still experience (10) recurrent stenosis even after undergoing effective reoperation. Recently, we encountered a patient who had previously undergone CAS, CEA and stent removal, and we successfully treated his third episode of carotid stenosis, which was caused by significant intimal thickening, through reCEA and patch angioplasty.

## Case description

All procedures performed in studies involving human participants were in accordance with the ethical standards of the institutional and/or national research committee(s) and with the Helsinki Declaration (as revised in 2013).

**Figure 1:**
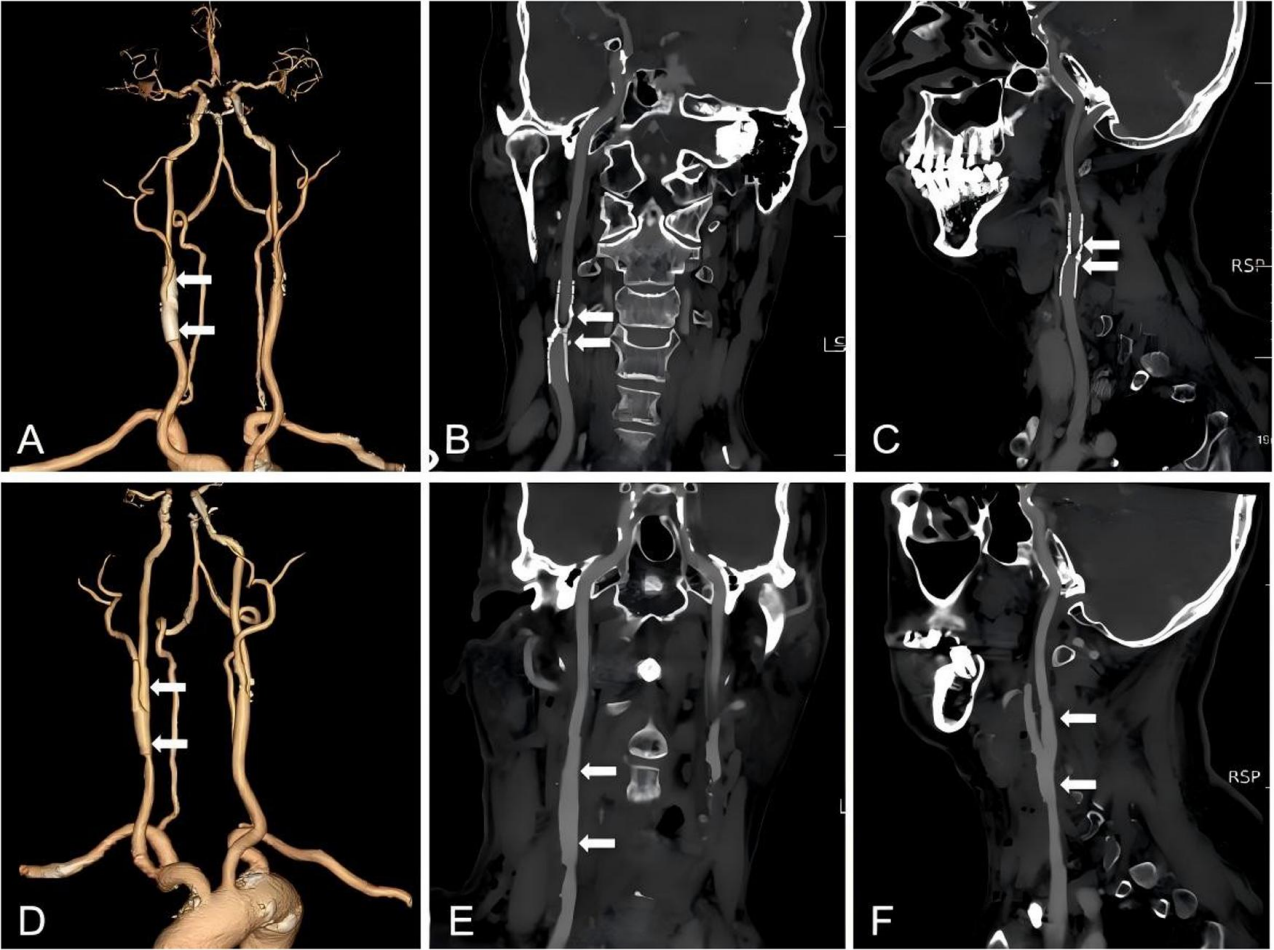
CTA images before and after the first CEA. Three-dimensional CTA images prior to CEA, and white arrows point to the location of the stent. **(A)** Coronal and sagittal CTA views show preoperative in-stent restenosis in the right internal carotid artery (white arrows). **(B–C)**. Three-dimensional CTA images following CEA combined with stent removal, and white arrows point to the site of stent excision. **(D)** Coronal and sagittal CTA sections show resolution of restenosis after operation (white arrows). **(E–F)**.

**Figure 2:**
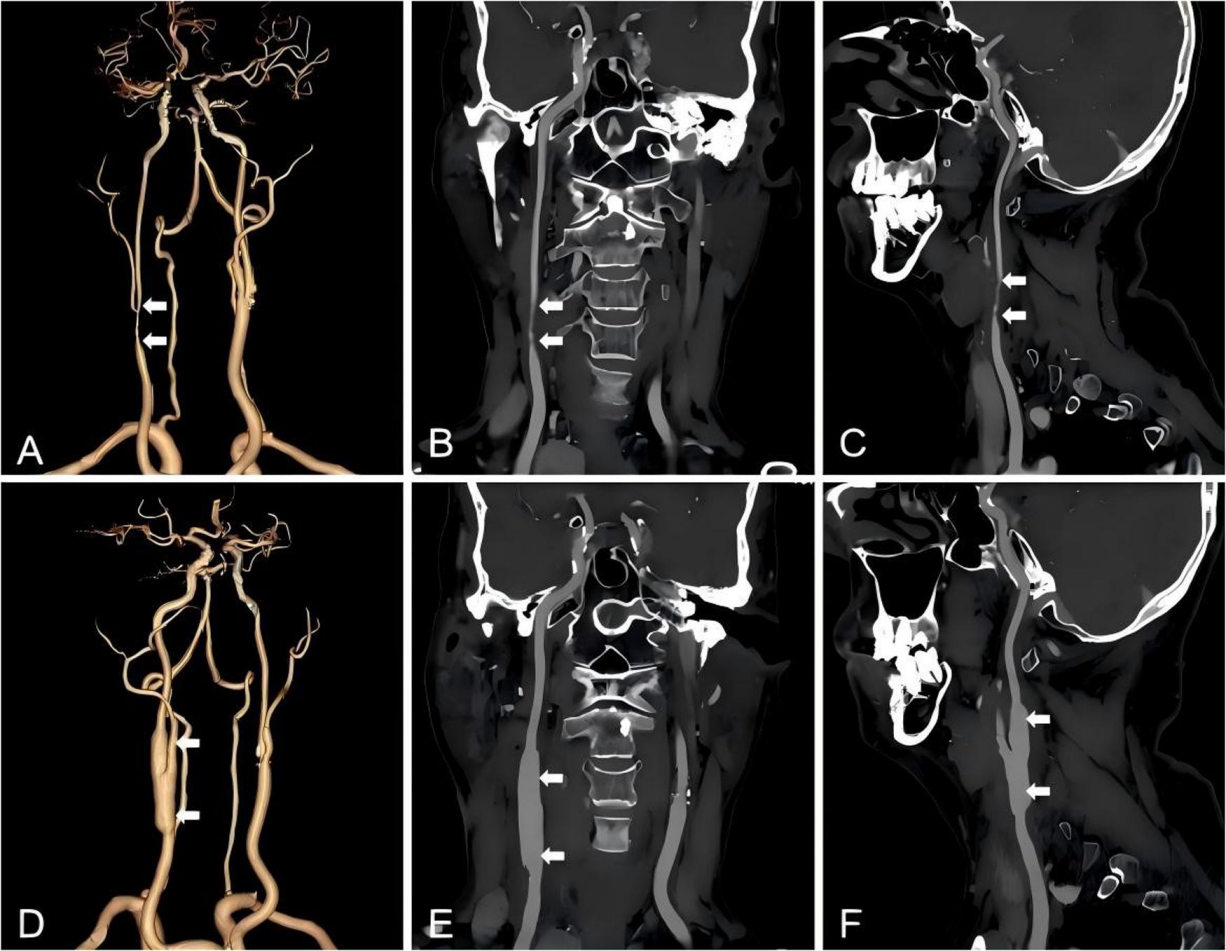
CTA images before and after the second CEA. Three-dimensional CTA images prior to reCEA, and white arrows point to the location of restenosis. **(A)** Coronal and sagittal CTA views show restenosis development in the right internal carotid artery following the initial CEA (white arrows). **(B–C)**. Three-dimensional CTA images following CEA combined with patch angioplasty, with white arrows pointing to the surgical site and the diameter of the blood vessels was increased. **(D)** Coronal and sagittal CTA sections show improved luminal patency and resolution of restenosis after operation (white arrows). **(E–F)**.

**Figure 3:**
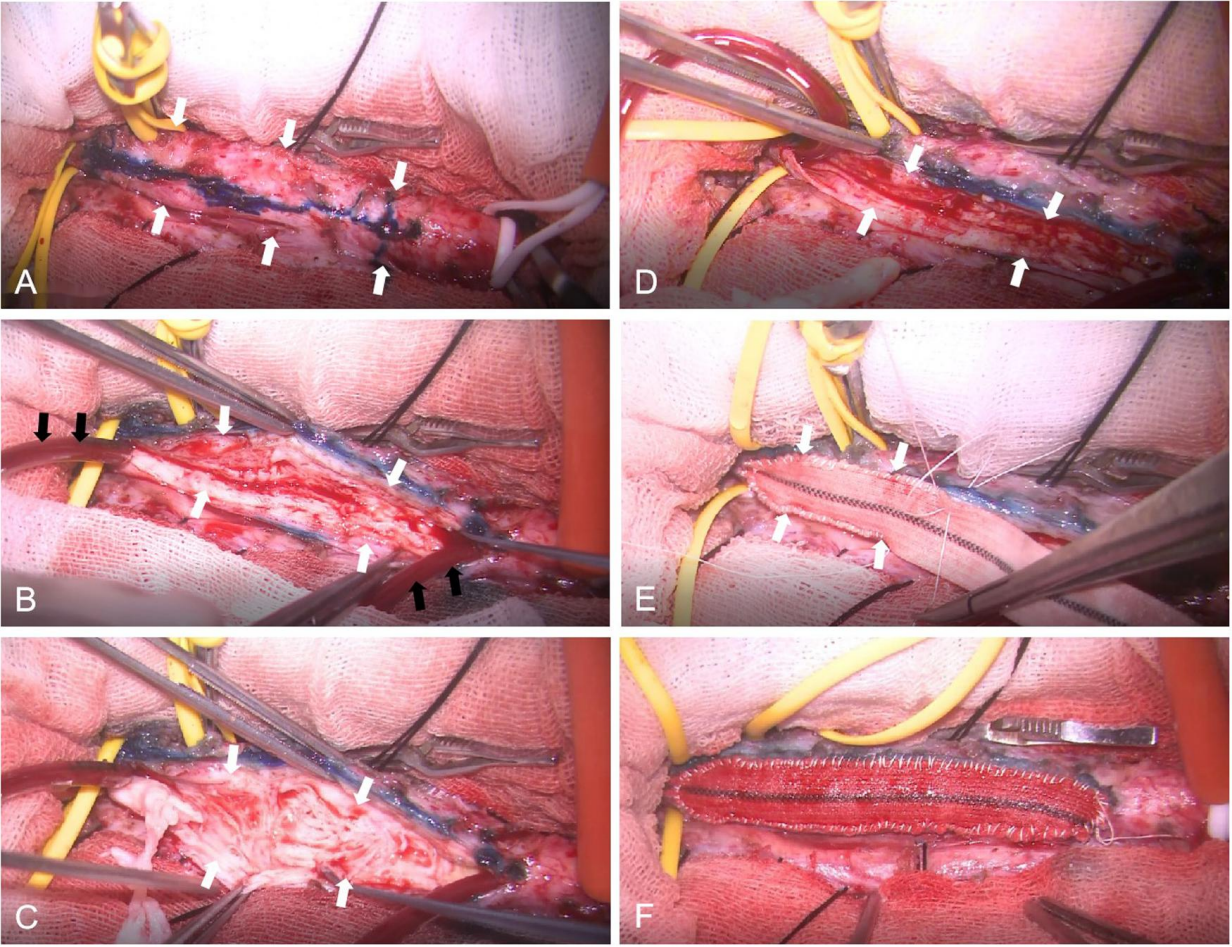
Intraoperative photograph of CEA combined with patch angioplasty. Exposure and temporary occlusion of the superior thyroid artery, external carotid artery, common carotid artery, and internal carotid artery. The white arrows point to the common carotid artery and internal carotid artery. **(A)** The anterior wall of the artery was incised, and a shunt was inserted (black arrows), with white hyperplastic intimal tissue observed within the arterial wall. (white arrows). **(B)** Dissection and removal of the thickened, fibrotic intimal (white arrows). **(C)** Appearance of the vascular lumen following completion of CEA (white arrows). **(D)** Patch angioplasty using a synthetic graft, with continuous non-absorbable suturing performed from proximal end. The white arrows point to the patch. **(E)** Following completion of operation, no bleeding or oozing is observed at the arterial wall and patch suture site. **(F).**

**Figure 4:**
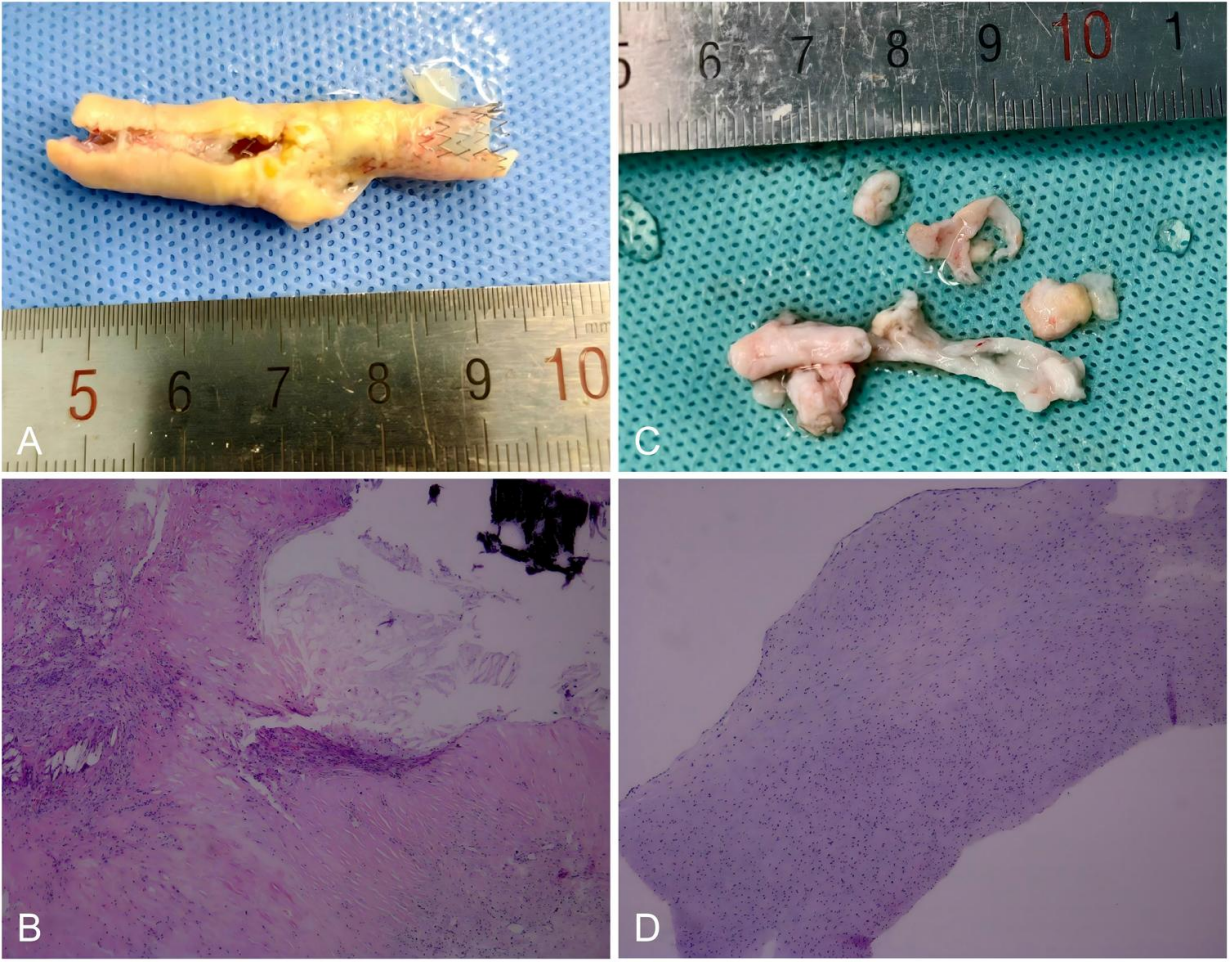
Gross and microscopic appearances of the plaque following two CEAs. Gross plaque image obtained after the first CEA, demonstrating a yellow plaque encircling the stent. **(A)** Histopathological image shows he presence of calcification, collagen fiber hyperplasia, hyalinization, inflammatory cell infiltration, and foam cells. Original magnification×50. **(B)** Gross plaque image obtained after the reCEA, demonstrating white fibrotic intimal tissue. **(C)** Histopathological image shows vascular smooth muscle cells in a background of myxoid degeneration and increased fibrosis without lipid component. Original magnification ×50. **(D).**

The 69-year-old man suffered a weakness and numbness of his left arm in May 2022, for which, at another hospital, right carotid artery stenting was performed. The patient has been taking 100 mg of bayaspirin and 20 mg of atorvastatin every day since 2022. Even during the stent surgery, an additional 75 mg of clopidogrel was taken daily. In May 2024, he developed symptoms of repeated dizziness, and the results of computed tomographic angiography (CTA) and ultrasound revealed 73% in-stent restenosis of the right internal carotid artery. CEA combined with stent removal was performed, as the diameter of the distal internal carotid artery was measured to be greater than 5 mm. Postoperative CTA demonstrated restored blood flow and successful alleviation of stenosis (Figure 1). In May 2025, the patient presented with recurrent transient ischemic attack (TIA); ultrasound demonstrated 78% diameter stenosis with a flow velocity of 393/161 cm/s, and CTA revealed a markedly narrowed carotid artery (Figure 2). A third surgical intervention was conducted on the restenosis carotid artery, which involved a technically challenging dissection. Upon exposure of the carotid artery, extensive white intimal hyperplasia was observed, nearly occluding the vascular lumen. The hyperplastic tissue exhibited a firm consistency, variable density, and an irregular surface, which collectively contributed to recurrent carotid stenosis. Following CEA, angioplasty was performed on the right internal using a synthetic patch to reduce the risk of late recurrence (Figure 3). A shunt was utilized to prevent cerebral ischemia during the period of vascular occlusion. The postoperative CTA revealed a successful alleviation of carotid artery stenosis, and the patient was discharged four days after surgery with complete neurological recovery (Figure 2). The pathological findings from the two time points are presented in Figure 4. A follow-up carotid ultrasound performed in August 2025 confirmed excellent vessel patency.

## Discussion

Stoney and String were the first to classify recurrent carotid lesions into two distinct types: lesions occurring within two years after CEA were attributed to intimal hyperplasia and thereafter to recurrent atherosclerosis (13). The similar point was made in subsequent articles (1, 5, 11). And there is also a view that intimal hyperplasia occurs within three years (2, 4). Due to the prolonged time interval, there appears to be a wide spectrum of changes ranging from lesions. Research has indicated that only 14%-19% were composed entirely of smooth muscle cells. In fact, the majority of lesions demonstrate both components intimal thickening and recurrent atherosclerosis, which can be can be identified both grossly and microscopically (4, 14). Restenosis occurring within 1 to 2 years of operations consisted of pale, firm, and homogeneous thickening of the intima with smooth luminal surfaces. Circumferential intimal thickening is considered to be part of the normal reparative process after CEA (15). However, unlike the case reported here, the thickened white intima almost completely occluded the vascular lumen within one year, resulting in the recurrence of vascular insufficiency. The mechanisms underlying intimal hyperplasia that lead to restenosis have not yet been fully clarified. Histologically, these lesions are predominantly composed of smooth muscle cells and extracellular matrix. It is hypothesized that, following intervention, the recently denuded surface is covered with platelets, fibrin, and entrapped red and leucocytes, which may stimulate smooth muscle cell proliferation, migration, and matrix formation (16–18). At present, agents that inhibit platelet aggregation and release have been widely used at the site of arterial injury to prevent intimal thickening.

At present, there is no unified standard for the treatment of carotid restenosis, especially for asymptomatic cases (3, 10, 19).The incidence of post-CEA stenosis has been reported to range from 1% to 36%, however, only 1% to 8% of all patients undergoing CEA have symptomatic hemodynamically significant recurrent carotid stenosis (1, 5, 7, 8, 20). Currently, surgical intervention is recommended for asymptomatic patients with carotid stenosis of ≥70% and for symptomatic patients exhibiting hemodynamic abnormalities (1, 2, 19). Historically, CEA has been regarded as the preferred treatment for carotid restenosis (21). In recent years, CAS has gained increasing attention in clinical research and has been proposed as an alternative to repeat surgical intervention for patients experiencing restenosis following CEA, particularly in cases of early (<24 months post-CEA) restenosis (10, 22, 23). This approach is based on the assumption that early intimal hyperplasia represents a relatively stable lesion, thereby posing a lower risk of distal embolism (1, 2). However, it is also associated with a higher incidence of in-stent restenosis of ≥50% (6, 9, 11). Other authors propose a carotid bypass for recurrent carotid stenosis (24). There are also studies reported restenosis resulting from intimal hyperplasia following CAS (16, 18, 21, 25).

Although the literature is replete with articles on the perioperative results of redo surgery (3, 6, 9, 10, 26–31), the pathological phenomenon of intimal thickening following CEA is rarely reported or only briefly discussed (5, 7, 26). In my view, this represents a topic of considerable significance. The recurrence of vascular stenosis in certain patients, despite undergoing multiple surgical interventions, may be attributed to both the composition of the plaque and the selection of the surgical approach. Edwards et al. found 3 patients with symptomatic recurrent stenosis to have “a tough, thick, fibrous lining” (32). In our case, following the opening of the carotid artery, it was observed that the thickened white intima nearly occluded the vascular lumen. The intimal surface was irregular, and the entire lesion exhibited a fibrotic, rigid consistency, making it challenging to achieve satisfactory restenosis prevention through standard CEA alone. We therefore performed carotid endarterectomy with patch angioplasty on the patient. Postoperative CTA demonstrated a markedly increased vessel diameter, which is expected to effectively prevent long-term restenosis. Also, It is difficult for the balloon to compress these tough and thick fibrous tissues, so that the stent cannot support the vessel with sufficient diameter, I believe that the CAS is not the best option for him. Currently, several studies have reported that redo CEA with patch angioplasty has yielded favorable outcomes in patients with restenosis following CEA (10, 15, 32, 33). W Yang even suggested all reCEA were performed using patches (34). However, only a limited number of studies have provided detailed pathological descriptions of intimal thickening (8, 35, 36).

## Key points of this operation

The skin and platysma muscle were incised longitudinally along the anterior border of the sternocleidomastoid muscle. The carotid sheath was carefully dissected to expose the common carotid artery, internal carotid artery, external carotid artery, and superior thyroid artery, while ensuring protection of the hypoglossal nerve. And then systemic heparinization is obtained. Subsequently, the superior thyroid artery, the external carotid artery, the common carotid artery, and the internal carotid artery adjacent to the hypoglossal nerve were temporarily occluded using arterial clamping clamps. The anterior wall of the common carotid artery and internal carotid artery was carefully incised using scissor, and a shunt was inserted to establish bypass circulation. White hyperplastic intima was observed within the arterial wall. The hyperplastic lesion was approximately 4.0 cm, extending from the distal segment of the common carotid artery to the proximal portions of both the internal and external carotid arteries. Given that the internal carotid artery diameter was less than 4 mm, patch angioplasty was performed. The arterial wall was closed using continuous 6-0 non-absorbable sutures. Prior to completing the anastomosis, the shunt balloon was deflated, and air and potential microthrombus were evacuated through the catheter. The final suturing was conducted after removal of the bypass cannula. Following the release of temporary occlusion, the artery exhibited strong pulsation. Until ensuring no evidence of bleeding or oozing blood at the arterial wall and patch suture site, carotid sheath, platysma muscle, subcutaneous tissue, and skin were closed in sequence.

## Conclusion

Symptomatic restenosis resulting from simple intimal hyperplasia within a short period following CEA is relatively uncommon. The intima hyperplasia tends to be thick, fibrotic, and irregular in morphology. To reduce the risk of recurrent stenosis, CEA combined with patch angioplasty may be considered as a preferred surgical approach for the management of these patients.

## Data Availability

The original contributions presented in the study are included in the article/Supplementary Material, further inquiries can be directed to the corresponding author.
